# Influence of cell distribution and diabetes status on the association between mitochondrial DNA copy number and aging phenotypes in the InCHIANTI study

**DOI:** 10.1111/acel.12683

**Published:** 2017-10-19

**Authors:** Ann Zenobia Moore, Jun Ding, Marcus A. Tuke, Andrew R. Wood, Stefania Bandinelli, Timothy M. Frayling, Luigi Ferrucci

**Affiliations:** ^1^ Longitudinal Studies Section Translational Gerontology Branch National Institute on Aging Baltimore MD USA; ^2^ Human Statistical Genetics Unit Laboratory of Genetics and Genomics National Institute on Aging Baltimore MD USA; ^3^ Genetics of Complex Traits University of Exeter Medical School Exeter UK; ^4^ Geriatric Unit Azienda Sanitaria di Firenze Florence Italy

**Keywords:** all‐cause mortality, cell distribution, copy number, diabetes, mitochondria, mitochondrial DNA

## Abstract

Mitochondrial DNA copy number (mtDNA‐CN) estimated in whole blood is a novel marker of mitochondrial mass and function that can be used in large population‐based studies. Analyses that attempt to relate mtDNA‐CN to specific aging phenotypes may be confounded by differences in the distribution of blood cell types across samples. Also, low or high mtDNA‐CN may have a different meaning given the presence of diseases associated with mitochondrial damage. We evaluated the impact of blood cell type distribution and diabetes status on the association between mtDNA‐CN and aging phenotypes, namely chronologic age, interleukin‐6, hemoglobin, and all‐cause mortality, among 672 participants of the InCHIANTI study. After accounting for white blood cell count, platelet count, and white blood cell proportions in multivariate models, associations of mtDNA‐CN with age and interleukin‐6 were no longer statistically significant. Evaluation of a statistical interaction by diabetes status suggested heterogeneity of effects in the analysis of mortality (*P *< 0.01). The magnitude and direction of associations between mtDNA‐CN estimated from blood samples and aging phenotypes are influenced by the sample cell type distribution and disease status. Therefore, accounting for these factors may aid understanding of the relevance of mitochondrial DNA copy number to health and aging.

## Introduction, results, discussion

Mitochondrial dysfunction is generally considered one of the hallmarks of aging (López‐Otín *et al*., 2013). However, robust evidence for the role of mitochondria in human health and aging is still lacking due to difficulty of assessing mitochondrial volume and function *in vivo* in large samples. Recently, relatively high‐throughput estimates of mitochondrial DNA copy number (mtDNA‐CN) have become feasible by quantitative PCR or extracting information from DNA microarray or sequencing data (Ashar *et al*., 2015; Qian *et al*., 2017). However, previous studies that attempted to relate mtDNA‐CN to phenotypes generally ignored technical factors that may contribute to variability in mtDNA‐CN and may lead to inconsistent and potentially biased estimates of association.

Mitochondrial content varies across tissue and cell types (Wachsmuth *et al*., 2016), and studies have shown that the distribution of different blood cells types (platelets, white blood cells, leukocytes subgroups) may significantly affect estimates of mtDNA‐CN in whole blood (Knez *et al*., 2016). The distribution of white blood cell subtypes changes systematically with aging and diseases. Together, these observations suggest that blood cell distribution may confound associations under investigation. Further, given that mtDNA‐CN estimation methods depend on the ratio of mitochondrial to nuclear DNA, the presence of enucleated cells (platelets) may also shift observations. Evaluation of heterogeneity of effects, potential effect modification, due to differences in the mechanisms that increase mtDNA‐CN is also warranted. Increases in mtDNA‐CN may represent an increase in mitochondrial biogenesis that is an appropriate and beneficial response to energetic demands or moderate stress. In contrast, it has been hypothesized that in the context of dysfunction or damage, mtDNA‐CN may capture a mixture of intact and fragmented mtDNA (Malik & Czajka, 2013). Current methods for high‐throughput estimation of mtDNA‐CN cannot distinguish between possible mechanisms from the observed value but stratification by diseases associated with mitochondrial damage/dysfunction such as diabetes (Szendroedi *et al*., 2012) may be informative.

Here, we explore the effect of these sources of variability and potential bias in 672 participants from the 6‐year follow‐up visit of the InCHIANTI study, a population‐based prospective cohort study of residents from two areas in the Chianti region of Tuscany, Italy (Ferrucci *et al*., 2000, Appendix S1). The mean age of the study sample was 70 years (range: 29–96), and 45.1% of participants were male (Table S1). The fastMitoCalc algorithm (Qian *et al*., 2017) was applied to low‐pass whole‐genome sequencing reads from buffy coat samples to estimate mtDNA‐CN (Appendix S1). The algorithm assumes DNA sequencing coverage is proportional to the underlying DNA copy number for autosomal and mitochondrial DNA; hence, the mtDNA‐CN per cell can be estimated by the following: (mtDNA average coverage/autosomal DNA average coverage)×2. The average estimate of mtDNA‐CN per cell across participants was 127.3 (SD: 29.4, range: 48.4–256.6). mtDNA‐CN varied by white blood cell composition, and there was a trend toward higher copy number among younger participants and women (Table S1, Fig. S1).

Coefficients from a series of multivariable linear regression and Cox proportional hazard models were used to describe the magnitude and direction of association between mtDNA‐CN and representative phenotypes. Age, interleukin‐6 (IL‐6), hemoglobin, and all‐cause mortality were selected as outcomes because of their importance to gerontology and potential association with mitochondrial function. Initial models were adjusted for age, sex, study site, smoking status, and autosomal coverage as appropriate. Then, platelet and white blood cell counts, as well as neutrophil, lymphocyte, monocyte, and eosinophil percentages measured in a complete blood count, were introduced as covariates in the initial models. After adjusting for blood cell type distribution, mtDNA‐CN coefficients estimating the association with age, IL‐6, and mortality were substantially attenuated; coefficients were no longer significantly different from zero in age and IL‐6 models (Table 1). On the contrary, the magnitude of the coefficient estimating the association between mtDNA‐CN and hemoglobin increased by 30%. By analogy, it is plausible that analyses that focus on other outcomes could be similarly affected by cell composition and lack of adjustment for such confounding may substantially alter the interpretation of results.

**Table 1:** Measures of association estimating the relationship between mtDNA‐CN and outcomes of interest among InCHIANTI study participants

			Model 1^a^		Model 2 Model 1 + white blood cell and platelet count		Model 3 Model 2 + neutrophil, lymphocyte, monocyte and eosinophil percent	
A	Outcome	*n*	β (SE)	*P*	β (SE)	*P*	β (SE)	*P*
Age, yrs		672	−1.15 (0.58)	0.049	−1.21 (0.60)	0.044	−0.86 (0.58)	0.137
log(IL6)		665	−0.07 (0.02)	0.002	−0.05 (0.02)	0.052	−0.03 (0.02)	0.200
Hemoglobin, g/dL		672	0.07 (0.05)	0.143	0.11 (0.05)	0.026	0.10 (0.05)	0.058
B	Outcome	*n* (events)	HR (95% CI)	*P*	HR (95% CI)	*P*	HR (95% CI)	*P*
Mortality		672 (210)	0.87 (0.76, 1.01)	0.070	0.89 (0.76, 1.03)	0.118	0.91 (0.78, 1.05)	0.196

a A: Multivariable linear regression models adjusted for age (except in the age model), sex, smoking status (never/former/current), study site, and autosomal coverage. B: Cox proportional hazard model adjusted for age, sex, smoking status (never/former/current), study site, and autosomal coverage.

The potential for a statistical interaction by diabetes status was evaluated with the addition of a diabetes main effect and an interaction term between mtDNA‐CN and diabetes status. In a survival analysis testing the association of baseline mtDNA‐CN with mortality, the presence of a significant interaction suggested that the association between mtDNA‐CN and death is statistically significantly different (*P* = 0.005) for nondiabetics and diabetics, and a consistent trend is observed for the association with hemoglobin (Table 2, Fig. S2). Noteworthy, directions of association are consistent with the idea that increased mtDNA‐CN in diabetics may reflect an aggregation of DNA from dysfunctional organelles and that multiple mechanisms may contribute to the observed distribution of mtDNA‐CN. Phenotypic information including disease status and health behaviors that influence mitochondrial biogenesis may be key to the evaluation of mtDNA‐CN in association studies.

**Table 2:** Measures of association estimating the relationship between mtDNA‐CN and outcomes of interest in models including terms for a statistical interaction with diabetes among InCHIANTI study participants

Parameter^a^			mtDNA‐CN, standardized		Diabetes		mtDNA‐CN × diabetes	
A	Outcome	*n*	β (SE)	*P*	β (SE)	*P*	β (SE)	*P*
Age, years		672	−0.75 (0.62)	0.225	8.13 (1.78)	<0.001	0.43 (1.56)	0.782
log(IL6)		665	−0.02 (0.03)	0.320	0.10 (0.07)	0.193	−0.02 (0.06)	0.756
Hemoglobin, g dL^−1^		672	0.14 (0.06)	0.013	0.14 (0.16)	0.403	−0.25 (0.14)	0.077
B	Outcome	*n* (events)	HR (95% CI)	*P*	HR (95% CI)	*P*	HR (95% CI)	*P*
Mortality		672 (210)	0.82 (0.69, 0.97)	0.024	1.69 (1.15, 2.46)	0.007	1.52 (1.14, 2.03)	0.005

a Other covariates in the multivariable linear regression models (A) and Cox proportional hazard model (B) include age (except in the age model), sex, smoking status (never/former/current), study site, autosomal coverage, white blood cell count, platelet count, neutrophil percent, lymphocyte percent, monocyte percent, and eosinophil percent.

To evaluate factors that may influence inference from analysis of mtDNA‐CN, we have used data from the InCHIANTI study, a population that is very well characterized but that is also geographically homogenous, moderately sized, and relatively healthy. Understanding of the issues raised would be improved through replication in other studies reflecting the range and complexity of mitochondrial function and aging phenotypes as well as variability in mtDNA‐CN measurement methods and sample tissue types. While further studies are warranted, the observations presented here highlight the importance of accounting for potential confounding by cell type and parsing potential differences in mechanism related to comorbid conditions in analyses of mtDNA‐CN and aging phenotypes.

## Funding

This work was supported by the Intramural Research Program of the NIH, National Institute on Aging. The InCHIANTI Follow‐up 2 and 3 studies (2004‐2010) were financed by the U.S. National Institute on Aging (Contract: N01‐AG‐5‐0002), supported in part by the Intramural Research Program of the National Institute on Aging, National Institutes of Health, Baltimore, Maryland. Whole‐genome sequencing was supported by Wellcome Trust grants: 083270/Z/07/Z and 090367/Z/09/Z.

## Author contributions

AZM analyzed, interpreted, and drafted the manuscript. JD, MAT, and ARW involved in bioinformatics and reviewed the manuscript. SB, TMF, and LF designed the study and reviewed the manuscript.

## Conflict of interest

None declared.

## Supporting information


**Appendix S1** Experimental procedures.
**Fig. S1** Distribution of white blood cell subpopulation proportions by mtDNA‐CN among InCHIANTI study participants in the study sample (*n* = 672).
**Fig. S2** Crude association between mtDNA‐CN and outcomes of interest among InCHIANTI study participants in the study sample stratified by diabetes status.
**Table S1** Characteristics of InCHIANTI study participants in the study sample (*n* = 672) by mtDNA‐CN category.
