# Supplementary material for: Influence of cell distribution and diabetes status on the association between mitochondrial DNA copy number and aging phenotypes in the InCHIANTI study
Source: Aging Cell. 2017 Oct 19;17(1):e12683. doi: 10.1111/acel.12683 (PMC5770782; doi:10.1111/acel.12683)

**Supporting Information**

**Experimental procedures**

*Study population*

The InCHIANTI study is a population based prospective cohort study of residents from two areas in the Chianti region of Tuscany, Italy. Selection of study participants and data collection procedures have been described previously (Ferrucci et al., 2000). InCHIANTI study participants were enrolled between 1998 and 2000, with four study visits through 2015. Samples for the present analyses were drawn from the 6-year follow-up visit (2004-2006). Whole-genome low-pass DNA sequencing was completed for 680 participants. The study population includes individuals with sequencing data meeting quality control criteria and reads aligned to the mitochondrial genome (n = 672). InCHIANTI study protocols were approved by the Instituto Nazionale Riposo e Cura Anziani institutional review board in Italy and study participants provided informed consent.

*DNA collection and whole-genome sequencing*

Low-pass whole-genome sequencing was applied to genomic DNA extracted from buffy coat samples for 680 participants of the InCHANTI study. Participants were selected based on available microarray based genotype data, gene expression, and biomarker data. Sequencing was accomplished using an Illumina HiSeq 2000 with a minimum read depth of 6× at the Beijing Genomics Institute. Participants with data suggestive of contamination or sample mismatch were excluded from analyses.

*mtDNA copy number*

mtDNA-CN was estimated using the fastMitoCalc algorithm (Qian et al., 2017). This algorithm is a faster version of the original *mitoCalc* algorithm proposed by Ding and colleagues (Ding et al., 2015) to estimate mtDNA-CN from whole-genome sequences. Both programs are based on the rationale that DNA sequencing coverage should be proportional to the underlying DNA copy number for autosomal and mitochondrial DNA, and hence the mtDNA-CN per cell can be estimated by: (mtDNA average coverage/autosomal DNA average coverage)×2. The factor 2 reflects the two copies of autosomal DNA in a cell. Sequencing overage was drawn from the aligned bam files: average mtDNA coverage is based on the full mitochondrial genome and average autosomal coverage is based on 3000 randomly selected autosomal DNA fragments of 1000bp in length. Across samples in the study population the ranges of estimated average autosomal coverage and mtDNA coverage were 5.8-12.9 and 145.4-1090.0 respectively. mtDNA-CN estimates from the mitoCalc algorithm have previously been demonstrated to correlate with qPCR based estimates of mtDNA-CN: a correlation coefficient of .82 was observed between methods in 18 samples randomly selected from the SardiNIA study (Ding et al., 2015).

Several quality control filters are applied in the fastMitoCalc/mitoCalc algorithms: mtDNA-CN estimates are based upon uniquely mapped paired end reads and all reads included in analyses have a Mapping Quality Score (MAPQ) >=20 (theoretical probability of incorrect alignment < 1%). Never the less a limited number of reads coming from nuclear mitochondrial sequences (NUMTs) could be mistakenly mapped to mtDNA inflating mtDNA-CN estimates. However, this inflation is likely to be uniform across participants: correlation between fastMitoCalc mtDNA-CN estimates and true mtDNA-CN will be extremely high and any inflation would have minimal impact on downstream analyses.

*Phenotypes of interest and other independent variables*

Fasting blood samples were collected during InCHIANTI study visits using standardized protocols. Hemoglobin, white blood cell count, platelet count, as well as the proportion of neutrophils, lymphocytes, monocytes, eosinophils and basophils were obtained from a complete blood count and hemochrome analysis using a Coulter LH 750 Hematology Autoanalyzer (Beckman Coulter Inc, Brea, CA, USA). IL6 was measured by ELISA using a commercial assay (Quantikine HS Human IL-6 Immunoassay, R&D Systems Inc, Minneapolis, MN, USA): values were natural log transformed for analyses.

Demographic and health behavior information including smoking status were obtained through a structured interview. Height, weight and other anthropometric measurements were assessed using a standardized protocol. Diabetes status was adjudicated based upon: fasting blood glucose ≥ 126, glycosuria, medication, or a combination nof self-report and diet. Vital status and dates of death were confirmed through a systematic search of municipality records through 2014. Follow-up time was calculated as time in years elapsed between date of baseline study visit and date of death or December 31, 2014. Individuals who emigrated were censored based upon date of last contact.

*Statistical analysis*

The distribution of demographic characteristics, biomarkers and cell count information across categories of mtDNA-CN were summarized using descriptive statistics. The association between mtDNA-CN and age was evaluated in a series of multivariate linear regression models: the base model included sex, study site, smoking status and autosomal coverage. A second model incorporated blood count variables, white blood cell count and platelet count; the final model also included neutrophil, lymphocyte, monocyte and eosinophil percent. In all analyses mtDNA-CN was standardized, age was centered at 70 years, and blood count variables were mean centered. Associations with log transformed IL6 and Hemoglobin were evaluated in similar series of regression models with an additional covariate for age. The relationship between copy number and mortality was estimated in Cox proportional hazard models with the same series of covariates. The potential for heterogeneity of effects by diabetes status was evaluated with the addition of a diabetes main effect and an interaction term between mtDNA-CN and diabetes status in fully adjusted regression models. All analyses were performed using R 3.2 (R Foundation for Statistical Computing, Vienna, Austria).

**References**

Ding et al., 2015Ding, J., Sidore, C., Butler, T. J., Wing, M. K., Qian, Y., Meirelles, O., Busonero, F., Tsoi, L. C., Maschio, A., Angius, A., Kang, H. M., Nagaraja, R., Cucca, F., Abecasis, G. R. & Schlessinger, D. (2015), ‘Assessing mitochondrial dna variation and copy number in lymphocytes of  2,000 sardinians using tailored sequencing analysis tools.’, *PLoS Genet* **11**(7), e1005306. http://dx.doi.org/10.1371/journal.pgen.1005306

Ferrucci et al., 2000Ferrucci, L., Bandinelli, S., Benvenuti, E., Di Iorio, A., Macchi, C., Harris, T. & Guralnik, J. (2000), ‘Subsystems contributing to the decline in ability to walk: bridging the gap between epidemiology and geriatric practice in the inchianti study.’, *J Am Geriatr Soc* 48(12), 1618–1625.

Qian et al., 2017Qian, Y., Butler, T. J., Opsahl-Ong, K., Giroux, N. S., Sidore, C., Nagaraja, R., Cucca, F., Ferrucci, L., Abecasis, G. R., Schlessinger, D. & Ding, J. (2017), ‘fastmitocalc: an ultra-fast program to estimate mitochondrial dna copy number from whole-genome sequences.’, *Bioinformatics (Oxford, England)* 33, 1399–1401.

**Supplementary Tables and Figures**

Table S1: Characteristics of InCHIANTI study participants in the study sample (n = 672) by mtDNA-CN category.

| Characteristic | Estimated mtDNA-CN category | | p† |
| --- | --- | --- | --- |
|  | < median* | ≥ median |  |
| Age, yrs, mean (SD) | 71.3 (15.5) | 69.3 (15.4) | 0.091 |
| IL6, pg/mL, mean (SD)‡ | 4.19 (3.35) | 3.45 (2.87) | 0.002 |
| Hemoglobin, g/dL, mean (SD) | 13.80 (1.58) | 14.02 (1.35) | 0.044 |
| Deceased, n (%) | 123 (36.6) | 87 (25.9) | 0.004 |
| Diabetes, n (%) | 47 (14.0) | 27 (8.0) | 0.019 |
| Male, n (%) | 160 (47.6) | 143 (42.6) | 0.215 |
| Smoking status, n (%) |  |  | 0.243 |
| Former | 110 (32.7) | 111 (33.0) |  |
| Current | 37 (11.0) | 51 (15.2) |  |
| White blood cell count, K/µL, mean (SD) | 6.52 (1.71) | 6.16 (1.70) | 0.007 |
| platelet count, K/µL, mean (SD) | 240.4 (58.0) | 247.7 (63.3) | 0.121 |
| Neutrophils, %, mean (SD) | 59.1 (8.8) | 57.0 (8.3) | 0.002 |
| Lymphocytes, %, mean (SD) | 29.7 (8.4) | 31.3 (7.8) | 0.012 |
| Monocytes, %, mean (SD) | 7.7 (2.1) | 7.8 (2.0) | 0.425 |
| Eosinophils, %, mean (SD) | 3.0 (1.9) | 3.3 (2.3) | 0.053 |
| Basophils, %, mean (SD) | 0.5 (0.2) | 0.6 (0.2) | <0.001 |

* Range for < median: 48.36 - 124.95, ≥ median: 124.97 - 256.60; n = 336 in each group

† t-test or Fisher’s exact test.

‡ n_IL6_ = 665

Figure S1: Distribution of white blood cell subpopulation proportions by mtDNA-CN among InCHIANTI study participants in the study sample (n = 672).


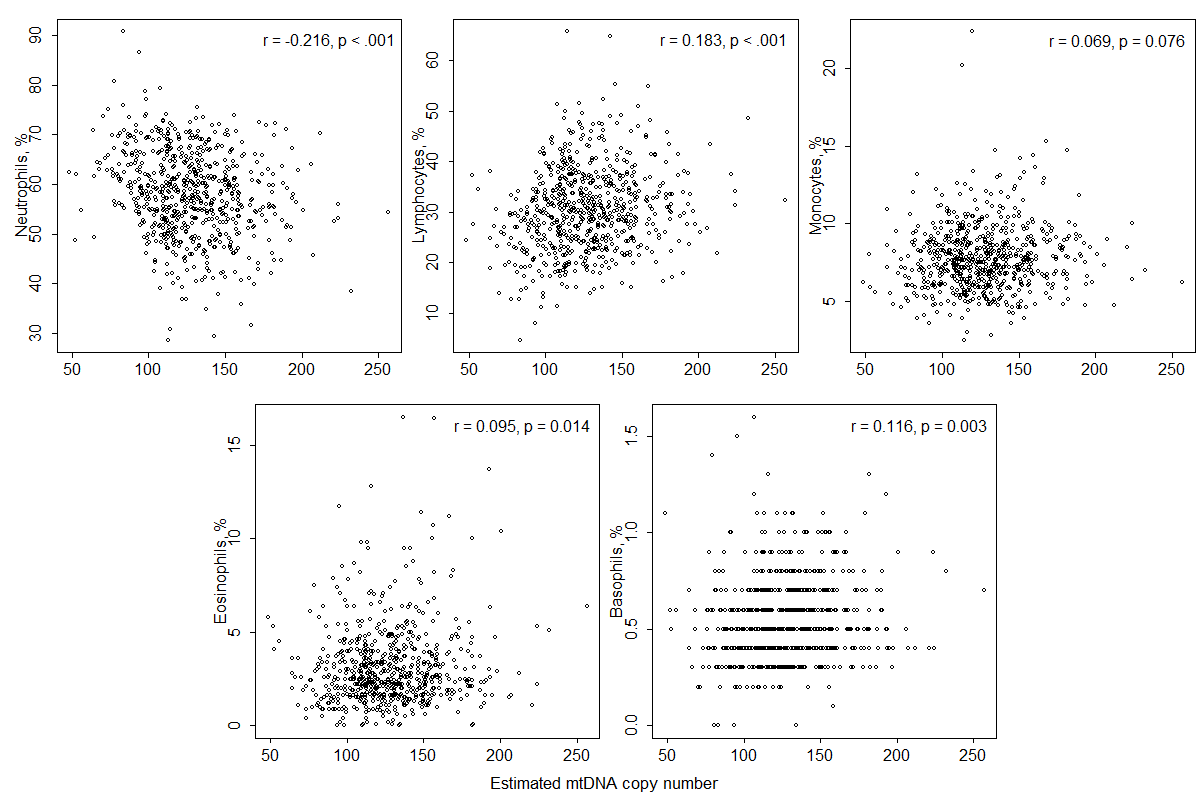


Figure S2: Crude association between mtDNA-CN and outcomes of interest among InCHIANTI study participants in the study sample stratified by diabetes status. A - Univariate linear regression of hemoglobin on continuous mtDNA-CN. B – Kaplan-Meier plot of survival after 6-year study follow-up by strata of mtDNA-CN.


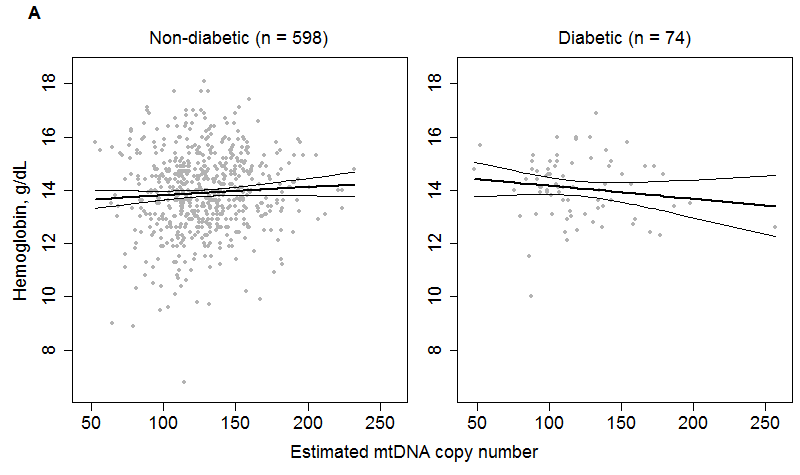


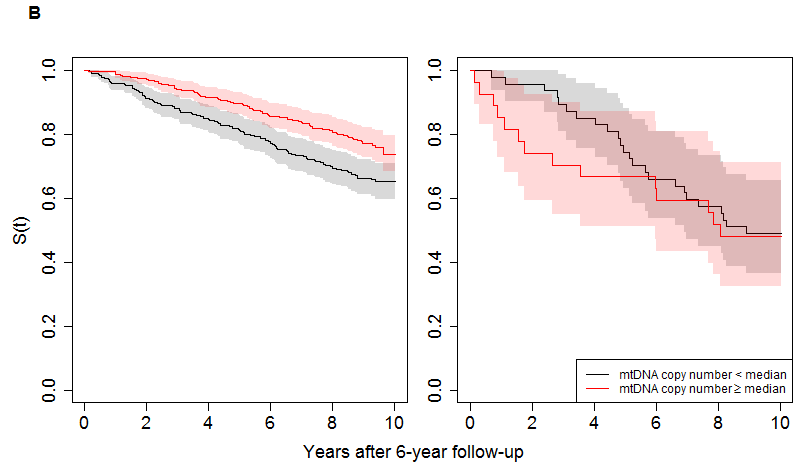

Supplement: Supplementary file 1 — Appendix S1 Experimental procedures. Fig. S1 Distribution of white blood cell subpopulation proportions by mtDNA‐CN among InCHIANTI study participants in the study sample (n = 672). Fig. S2 Crude association between mtDNA‐CN and outcomes of interest among InCHIANTI study participants in the study sample stratified by diabetes status. Table S1 Characteristics of InCHIANTI study participants in the study sample (n = 672) by mtDNA‐CN category. [file ACEL-17-na-s001.docx]
